# Determinants of Cycling Performance: a Review of the Dimensions and Features Regulating Performance in Elite Cycling Competitions

**DOI:** 10.1186/s40798-020-00252-z

**Published:** 2020-06-03

**Authors:** Kathryn E. Phillips, William G. Hopkins

**Affiliations:** Institute for Health and Sport, College of Sport and Exercise Science, Victoria University, Melbourne, Australia

## Abstract

**Background:**

A key tenet of sports performance research is to provide coaches and athletes with information to inform better practice, yet the determinants of athletic performance in actual competition remain an under-examined and under-theorised field. In cycling, the effects of contextual factors, presence of and interaction with opponents, environmental conditions, competition structure and socio-cultural, economic and authoritarian mechanisms on the performance of cyclists are not well understood.

**Objectives:**

To synthesise published findings on the determinants of cyclists’ behaviours and chances of success in elite competition.

**Methods:**

Four academic databases were searched for peer-reviewed articles. A total of 44 original research articles and 12 reviews met the inclusion criteria. Key findings were grouped and used to shape a conceptual framework of the determinants of performance.

**Results:**

The determinants of cycling performance were grouped into four dimensions: features related to the individual cyclist, tactical features emerging from the inter-personal dynamics between cyclists, strategic features related to competition format and the race environment and global features related to societal and organisational constraints. Interactions between these features were also found to shape cyclists’ behaviours and chances of success.

**Conclusion:**

Team managers, coaches, and athletes seeking to improve performance should give attention to features related not only to the individual performer, but also to features of the interpersonal, strategic, global dimensions and their interactions.

## Key Points

- The challenges of accurately modelling the performance of elite cyclists in complex racing environments using the traditional reductionist approach become evident when the features and dimensions influencing race performance are collated.
- Cyclist performance is constrained by features related to the individual cyclist, tactical features emerging from the inter-personal dynamics between cyclists, strategic features related to the competition, and global features related to the organization of the sport.
- Sports performance researchers need to find methodologies and techniques that enable elements of performance to be considered in concert rather than in isolation, and for the complex interplay and interactions between dimensions and features of racing to be better understood.

## Introduction

The focus of sports performance research is to provide coaches and athletes with information to inform better practice, yet the dimensions and features shaping the performances of elite athletes in actual competitions remain under-examined and under-theorised [1, 2]. In elite cycling, the factors related to achieving success have been investigated predominantly using the traditional reductionist paradigm, where components of performance are isolated and examined in laboratories or solo time trials in order to reduce the influence of confounding variables. The physiological, biomechanical, nutritional, aerodynamic and physical components of elite cycling performance have all been examined from this perspective [3–6]. These investigations have identified an extensive number of features governing the performance of individual cyclists, but the interplay between these features is not well understood, and there is still a limited understanding of how cyclists regulate their behaviour in competitive performance environments [7–10].

In elite cycling, which we define as competitions sanctioned by the Union Cycliste Internationale (UCI), the majority of competitions are race events, where opponents compete simultaneously to be first across the finish line. Characterised by the direct interaction permitted between opponents, race events can range from one-versus-one to mass-start contests and are found in most cycling disciplines (road, track, BMX, cyclo-cross and mountain biking). The proximity of opponents in racing contests results in behavioural dynamics not evident in solo performances, as riders constantly adapt to the actions of their opponents and the changing structure of the race environment [11–13]. The contextual, temporal, and spatial parameters shaping athlete behaviour must be better understood if we are to further our knowledge of the determinants of cycling performance in race events.

In recent decades, there has been an increased recognition of the complex interplay between features regulating an athlete’s competitive performance and a call for research that takes an integrated approach to the study of athlete behaviour [14–17]. Seifert and colleagues [14] argued for the need to study athlete behaviour at different levels of analysis, recognising athletes as complex adaptive systems whose behaviour is governed by their interaction with opponents, teammates and the specific constraints of the performance context. The aim of the current project was to synthesise findings from existing academic literature to build an integrated understanding of the dimensions and features underpinning cyclists’ behaviours and chances of success in elite racing.

In the interest of reflexivity (transparency about the perspectives of the authors), the primary author worked within a national elite cycling program for close to a decade. Her experience of the limitations of the existing scientific literature in addressing the complex interrelatedness of elite performance helped shape the design of this study and elements of the interpretation. Professional experience and knowledge provide a valuable lens through which to examine the research [18], and narrative synthesis enables a wide range of research to be systematically reviewed and synthesised [19]. Her co-author has more than 20 years of experience with quantitative assessment of athletic performance.

## Methods

### Literature Search

Four academic databases (PubMed, ScienceDirect, SPORTDiscus, Google Scholar) were searched for peer-reviewed articles related to the study of cycling performance in elite competition. Search terms initially included combinations of the following keywords: athlete, bicycling, competition, contest, elite cycling, performance, peloton, professional cycling, road racing and track cycling. Using the reference lists of these primary identified articles, an additional snowball search was undertaken, with further database searches conducted using additional search terms and highly relevant articles added [20]. The terms added were as follows: BMX, cyclo-cross, Giro de Italia, Grand Tour, mountain-biking, road cycling, time-trial, Tour de France, world class and Vuelta a España. Following this widened search, the titles and abstracts of all articles were reviewed, duplicates removed, and 139 papers identified for potential inclusion.

### Inclusion Criteria

**Fig. 1:**
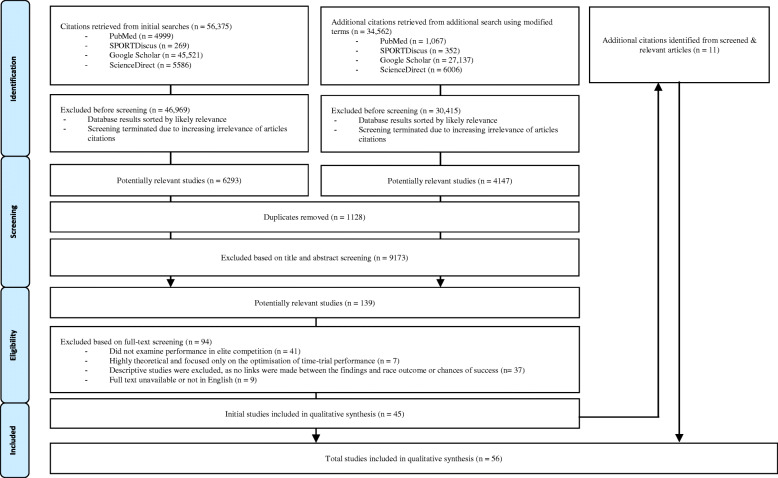
Flow diagram of the screening process. See ‘Inclusion Criteria’ section for the criteria defining the initial and modified searches

Research articles had to meet the following criteria to be eligible for inclusion: analyses were of cyclists at the elite level (those who had performed in competitions sanctioned by the UCI), authors sought to identify and explain the determinants of performance in actual competitions, and articles were published in peer-reviewed journals or books in English with full text available. In total, 44 original research and 12 reviews met the inclusion criteria. Of the excluded publications, 41 articles did not examine performance in elite competition, seven were modelling or mathematics papers that were highly theoretical and focused only on the optimisation of time-trial performance, and a further 37 descriptive studies were excluded, as no links were made between the findings and race outcome or chances of success. The studies selected for final inclusion incorporated research with mathematical, physiological, psychological, sociological, management, economic and game theoretical approaches. Thirty-seven research articles focused on the performance of elite cyclists in professional road racing, with a further nine articles focused on track cycling, three on mountain biking, one on cyclo-cross and six articles examined a mix of cycling disciplines (see Fig. 1).

### Data Extraction and Study Interpretation

A narrative synthesis approach was taken to systematically review the included articles, as this allowed synthesis of findings from multiple studies with considerable heterogeneity in their methods, participants, cycling discipline and theoretical underpinnings. In a preliminary synthesis, the key characteristics of all relevant articles were captured, including the following: author(s), date, title, discipline of cycling, methodology, competition level, sample size, aim of study, variables used, key findings and conclusions. The key findings from the selected studies were then grouped according to the setting or context of the investigation and according to the nature of the feature(s) being reported (see Table 1). These groupings were used to construct a rubric that helped explain the determinants of cycling performance, as presented below. The relationships within and between these groupings were also explored. A standardised risk-of-bias assessment was unable to be conducted due to the heterogeneity in study designs across the included articles, but a critical review of methodological quality is addressed where necessary.

**Table 1:** Key characteristics of research articles investigating the performance of elite cyclists in competition, organised by level of analysis and highlighting the performance features focused on in each study

Level of analysis	Cycling discipline	Method	Subjects and data	Performance features
The individual dimension				
Lucia et al. [21]	Road (time-trial)	Empirical analysis	11 professional road cyclists, 3 Tour de France time trial performances over 2 years (1998/1999)	Physiological features
Padilla et al. [22]	Road cycling	Empirical analysis	24 professional road cyclists	Physiological and morphological features (*links to competition features*)
Rodriguez-Marroyo et al. [23]	Road cycling	Empirical analysis	Workload demands on 30 professional road cyclists across 5-day, 8-day, and 21-day stage races (*n* = 10, 5 and 5 respectively) collected over 5 consecutive racing seasons.	Links between: physiological and competition features (*links also to team dynamics*, *team hierarchy*)
Impellizzeri et al. [24]	Mountain biking	Empirical analysis	12 internationally competitive cyclists, competing in one international level mountain bike race	Physiological features (*links to competition features*)
Impellizzeri et al. [25]	Mountain biking	Empirical analysis	13 male regional, national and international U23 cross-country mountain bike cyclists competing in a national level cross-country competition	Physiological and additional individual features ( *ability/experience level*) (*links to competition features*)
Chidley et al. [26]	Mountain biking	Mixed methods	Multiple study project. In study 3: 43 male cyclists ranging from junior, senior, master, expert and elite downhill mountain-biking categories	Physiological, cognitive, and additional individual features (*skill*, *self-confidence*)
Svendsen et al. [27]	Road cycling	Empirical analysis	Retrospective categorisation of 80 competitive male cyclists, including 9 World Tour cyclists	Physiological and additional individual features (*training, race experience*)
Moran and Pitsiladis [28]	Road cycling	Review	Review article	Additional individual features (*genetics and performance*)
Impellizzeri et al. [29]	Road cycling and mountain biking	Empirical analysis	27 professional female road cyclists and 12 elite female mountain bikers from eight different countries	Morphological and physiological features (*links to competition features*)
Dorel et al. [30]	Track cycling	Empirical analysis	12 male elite cyclists competing at National and International level track races	Physiological, morphological and additional individual features (*frontal surface area*, *optimal pedalling rate*)
Haake et al. [31]	Track cycling and road cycling	Empirical analysis	World records for the 1-h distance from 1894 to 1996, and for the 4-km individual pursuit from 1964 to 1996	Other individual features
Spindler et al. [32]	Various cycling disciplines	Review	Review article	Cognitive features
The tactical dimension				
Waldron et al. [33]	Track cycling	Empirical analysis	1 single race (24 riders) of international competitive cyclists (World Championship level)	Drafting and interpersonal features
Menaspà et al. [34]	Road cycling	Empirical analysis	Single-case study longitudinal design, retrospective analysis of one male professional road cyclist in the sprint finishes of 31 grand tour stages from 2008 to 2011	Drafting features, inter-personal features, team dynamics, competition features
Bossi et al. [35]	Cyclo-cross	Empirical analysis	329 cyclists (men + women) competing in 5 editions of the UCI World Championships (2012–2016)	Drafting features, competition features (weather)
Hoenigman et al. [36]	Road cycling	Agent-based modelling	The model ran 1800 trials of various combinations of cyclist strength and best strategy	Drafting and interpersonal features (*links to competition features*, *additional individual features*)
Trenchard [37]	Road cycling	Economic modelling	Theoretical analysis—modelling estimates based on drafting data from prior research	Drafting and interpersonal features (*links to physiological features*)
Trenchard [38]	Road cycling	Economic modelling	Two test protocols run with various levels of cyclists and adjusted variables	Drafting and interpersonal features (*links to physiological features*)
Scelles et al. [39]	Road cycling	Empirical analysis	268 breakaways, over 76 stages, 4 Tour de France events. Results were also bootstrapped.	Interpersonal and contextual features (*links to individual dimension*, *drafting and competition features*)
Dilger and Geyer [11]	Road cycling	Empirical analysis	49 sprint finals in which a small group of cyclists sprinted for the stage win. 26 duels, 13 three-ups, 10 finishes with between four and seven cyclists. 140 riders in total.	Drafting and interpersonal features (*links to individual dimension*)
Moffatt et al. [40]	Track cycling	Logistic regression models	231 races at 4 UCI World Cup competitions	Drafting, interpersonal and contextual features
Dwyer et al. [41]	Track cycling	Machine learning	4 races from World Cup (3) and World Championship (1) events across 1 season, incl. 91 cyclists (66 unique)	Drafting and contextual features
The strategic dimension				
Phillips and Hopkins [42]	Track cycling	Empirical analysis	336 UCI World Cup/World Championship and Olympic level cyclists (196 male, 140 female)	Competition features (*links to individual and tactical dimensions*)
Lucia et al. [43]	Road cycling	Empirical analysis	13 professional road cyclists, 8 ‘climbers’ and 6 ‘time trialists’ who had a stage win in a UCI event in the prior 2 years.	Link between competition features and individual dimension (physiological and morphological features)
Ofoghi et al. [44]	Track cycling	Machine learning	7 events, all cyclists, mix of 5 and 6 event omniums	Competition features (*links to individual dimension*)
Ofoghi et al. [45]	Track cycling	Machine learning	193 male omnium records, 167 female omnium records across 5 and 6 event omniums	Competition features: competition structure
Ofoghi et al. [46]	Track cycling	Empirical analysis	96 data records (men) and 75 data records (women) from four competitions, encompassing elite and junior racing at World Championship and National Championship level	Competition features (*links to individual dimension*)
Filipas et al. [47]	Road cycling	Empirical analysis	43 professional cyclists who achieved a top 10 pacing in a Grand Tour between 2010 and 2015	Links between team hierarchy, competition features and competition calendar
Larson and Maxcy [48]	Road cycling	Empirical analysis	All mass start stages of the three Grand Tours (1985–2010) *n* = 1436	Contextual features (*links to authoritarian and interpersonal features*)
Rodriquez-Gutierrez [49]	Road cycling	Empirical analysis	All professional cyclists belonging to the 18 UCI Pro teams in the year 2011. Sample equalled 448 cyclists.	Team features: opportunity (*links to morphological features*, *additional individual features, competition calendar features*)
Larson and Maxcy [50]	Road cycling	Modelling	Expansion of the model of Candelon and Dupuy [51] to incorporate coaching and production functions.	Team features, contextual features (*links to authoritarian features*, *economic features*, *reward mechanisms*)
Cabaud et al. [52]	Road cycling	Review	Method used on two 2014 Tour de France stages	Objectives and rewards, economic features, reward mechanisms and interpersonal features
The societal and organisational dimension				
Perneger [53]	Road cycling	Empirical analysis	5th place Grand Tour finishers from 1990 to 2009	Strategic dimension: competition features societal dimension: historical features (*links both to subversive behaviours*)
Lippi et al. [54]	Road cycling	Empirical analysis	Winners of Grand Tours since inception	Strategic dimension: competition features societal dimension: historical features (*links both to subversive behaviours*)
Rogge et al. [55]	Road cycling	Data envelopment analysis	31 cycling teams competing in the Tour de France over the period 2007–2011 (105 observations)	Team features and reward mechanisms (*links to competition and individual features*)
Rebeggiani and Tondani [56]	Road cycling	Empirical analysis	One season of Pro Tour racing data (2005)	Authoritarian and economic features (*links to competition features and reward mechanisms*)
Aubel et al. [57]	Road cycling	Discrete time-logit model	Data from 10,551 cyclists in the first three world divisions of cycling from 2005 to 2016, including 271 sanctioned cyclists	Subversive behaviours (*links to authoritarian, economic and reward mechanisms*)
Zheng 2016 [58]	Various	Interviews and document analysis	4 semi-structured interviews with lead Chinese staff + comprehensive document analysis	Authoritarian and economic features
Lodewijkx and Brouwer [59]	Road cycling	Empirical analysis	Winners of Grand Tours since World War 2	Social mechanisms and subversive behaviours (*links to authoritarian, economic and inter-personal features*)
Prinz and Wicker [60]	Road cycling	Socio-cultural analysis	Longitudinal dataset from Tour de France from 2004 to 2013 (1542 observations from 598 different cyclists)	Authoritarian features, team features (*links to individual features*)
Mignot [61]	Road cycling	Review	Theoretical analysis	History and prestige, economic and authoritarian features, competition calendar features
Fink and Smith [62]	Road cycling	Socio-cultural analysis	Socio-cultural examination of unofficial norms with examples given from previous editions of the Tour de France	Social mechanisms (*with links to authoritarian, economic and reward mechanisms*, *and subversive behaviours*)
Schumacher et al. [63]	Road and track cycling	Retrospective analysis	Race results of 4432 cyclists from 77 countries who had participated in major elite or junior elite cycling races from 1980 to 2004	Authoritarian features (*links to individual features*)
Larson and Maxcy [48]	Road cycling	Empirical analysis	Finishing position and relative times for top-25 cyclists finishing all mass-start stages of the three Grand Tours from 1985 to 2010 (*n* = 1436)	Authoritarian features (*with links to drafting, interpersonal and economic features*)
Brewer [64]	Road cycling	Review	Review article	Subversive behaviour and authoritarian mechanisms, history and prestige (*links to economic features*)
Multi-dimensional				
Albert [65]	Road cycling	Empirical analysis	Various: personal experience from racing, formal interviews with US riders and officials, cycling press and video reports. Senior men’s road racing in all instances.	Tactical dimension: drafting, interpersonal and contextual features Societal dimension: social mechanisms and authoritarian features (*all interlinked*)
Van Reeth and Larson [66]	Road cycling	Various levels	Review: comprehensive book with chapters exploring the various dimensions and features of professional road cycling—predominantly from an sports economics perspective.	Individual dimension: physiological, morphological, cognitive and additional features Tactical dimension: drafting, interpersonal and contextual features Strategic dimension: competition features, calendar effects, objectives, collusion and association, team features. Societal dimension: history and prestige, authoritarian, economic mechanisms, subversive behaviour (*various links*)
Torgler [67]	Road cycling	Ordered probit and ordinary least squares modelling	Tour de France, full data—21 teams, 188 cyclists (147 finishers)	Individual dimension: morphological features, experience effects, cultural background effects Tactical dimension: teammate effects Strategic dimension: opportunity effects
Mujika and Padilla [68]	Road cycling	Review	Review article	Individual dimension: physiological, morphological features Strategic dimension: competition features
Netland et al. [69]	Road cycling	Socio-cultural analysis	Semi-structured interviews: 9 persons (6 athletes, 3 directors) of Norwegian continental cycling teams. Plus 1 director and 3 cyclists from a different professional team.	Strategic dimension: team features Societal dimension: authoritarian, reward and social mechanisms
Lucia et al. [70]	Road cycling	Review	Review article	Individual dimension: physiological, morphological and additional individual features Strategic dimension: competition features (*and links to physiological and drafting features*) Societal dimension: subversive behaviours *(and links to additional individual features)*
Martin et al. [71]	Various disciplines (sprint finishes)	Review	Review article	Individual dimension: physiological features Tactical dimension: drafting features *(links to physiological features)*
Craig and Norton [5]	Track cycling	Review	Review article	Individual dimension: physiological and morphological features Strategic dimension: competition features (*links to physiological features*)
Santalla et al. [72]	Road cycling	Review	Review article	Individual features: physiological, morphological and additional individual features Strategic dimension: competition features (*links to physiological features*) Societal dimension: history and subversive behaviour features (*links to physiological features*)
Castronovo et al. [73]	Road cycling	Review	Review article	Individual dimension: physiological and cognitive features Strategic dimension: competition features (*links between these features*)
Menaspa and Abbiss [74]	Road cycling	Review	Review article	Individual dimension: physiological and morphological features Strategic dimension: competition features (*and the links between these*)

## Results and Discussion

The performances of elite cyclists in competitive elite racing are influenced by features related to the following four dimensions: the individual dimension and features related to the individual cyclist, the tactical dimension and features which emerged from the inter-personal dynamics between performers, the strategic dimension and features related to the competition format and environment, and the global dimension related to societal and organisational features of the sport. An overview of each dimension is presented below, identifying the key features and how these interact to shape cyclists’ behaviours and chances of success in elite racing.

### The Individual Dimension

Various features have been shown to govern the performance of individual cyclists in elite competitions, including the cyclists’ physiological and morphological features, cognitive skills, nationality, aptitude for risk, and physical attractiveness. Research in the field of sport and exercise science has tended to focus at this level of analysis [75].

#### Physiological Features

An individual cyclist’s performance has been attributed to their peak and functional power outputs, cardiovascular, pulmonary and other physiological capacities (see [3–6, 26, 68, 70, 72] for comprehensive reviews). In recent decades, the contribution of submaximal physiological variables, including cycling economy or efficiency, has also been highlighted [76–79]. Despite extensive study of cyclists’ physiological features, the extent to which these predict competitive success in races at the elite level is limited, particularly in mass-start racing [21, 25, 77, 80]. Impellizzeri and colleagues [24] found only 40% of the variance between international cross-country cyclists’ finishing times was explained by differences in physiological characteristics. Chidley and colleagues [26] found that most of the variance in finish times of downhill mountain bikers was explained by skill (*r*^2^ = − 0.76), with only a small proportion attributable to physiological variables (anaerobic capacity, *r*^2^ = 0.0; VO_2_max, *r*^2^ = − 0.3). Phillips and Hopkins [42, 81] demonstrated the tenuous links between an individual’s physiological features and elite competitive performance, finding that factors determining consistent individual time-trial performance transferred minimally to performance in mass-start or one-versus-one racing. Cyclists must have high levels of physiological fitness to reach the elite level, but at this level there appear to be little differences in their physiological characteristics, and therefore other factors contribute to race outcomes [25, 80]. Contextual, temporal and spatial parameters are also known to alter the physiological demands on a cyclist and their association with success. For example, the format and structure of a cycling competition alter the technical skill requirements and physiological demands on a cyclist, predisposing individuals with particular physiological features to be better suited for certain competition formats or races [22, 41, 43, 47, 78, 82, 83]. These links are highlighted further in ‘Competition Features: Terrain, Environment and Competition Structure’ section.

#### Morphological Features

A cyclist’s height, body weight, muscle size, and fibre composition have been shown to influence performance in specific types of races at the elite level [29, 30, 61, 67, 84]. Furthermore, the morphological characteristics of elite cyclists vary between cycling disciplines [5, 23, 29, 72], consistent with cyclists specialising into particular disciplines or team roles based on their morphology [85]. For example, road cyclists are frequently categorised according to their morphological characteristics as climbers, sprinters, or domestiques. As air resistance is one of the dominant forces a cyclist must contend with, certain morphological trade-offs occur. Having a greater muscle mass can enable a cyclist to better generate the energy and power required to overcome the drag caused from riding at high speeds on flat surfaces [84] but can also lead to an increased frontal surface area, thereby increasing drag and negatively affecting performance [30]. In the hill and mountain stages of professional road races, when speeds are low, overcoming gravity becomes more important than overcoming drag, and cyclists with low body mass tend to be more successful [22, 84]. These and other well-established links between a cyclist’s morphology, competition features and chances of success, are detailed further in ‘Competition Features: Terrain, Environment and Competition Structure’ section.

#### Emotional and Cognitive Features

The role of a cyclists’ cognitive features in regulating their behaviour and competition performance was highlighted by Spindler and colleagues in a recent review of the psychology of elite cycling [32]. A cyclist’s mood, levels of anxiety, self-confidence, ability to manage pain, attentional focus and cognitive function were identified as influencing performance, although the authors noted that the differing aims and objectives of the studies reviewed made it difficult to establish strong support for any particular association. There was tentative evidence that anxiety impairs performance amongst elite male road riders, that implicit beliefs affect decision-making performance, and that pain management is important to achieve success in elite road cycling [32]. There was also some evidence that the sex of a cyclist modified the effect of confidence on performance, and evidence of a difference in cognitive abilities between elite and sub-elite riders [32], but whether these cognitive differences predict success at the elite level remains to be seen. The potential influence of stress, mental fatigue, personality, and an individual’s implicit beliefs in regulating their behaviour and competition performance were also highlighted [32].

Larson and Macxy [80] provided further support for the importance of cognitive skills, explaining that cyclists must manage their energy expenditure, effort and pacing, optimise their aerodynamics in relation to their opponents, and make decisions not only in competition but also in training. Castronovo and colleagues [73] explored how cognitive features influence a cyclist’s perceived level of exertion and subsequent regulation of effort over the course of a race. Cyclists are known to subjectively distribute their energetic resources across the race to improve their performance, a strategy referred to as pacing [5, 86]. Cyclists adjust their pace according to their anticipated and accumulated levels of fatigue, terrain characteristics, race duration, elapsed distance, and competition structure [23, 35]. The pacing strategy a cyclist adopts is also known to be affected by the presence and behaviour of their opponents, and there is some evidence of a sex effect [23, 35, 87]. For example, Bossi and colleagues [35] found male and female cyclo-cross athletes distributed their efforts differently across the course of a race, but were unable to conclude whether this was due to differences in contextual features, such as race length, or psychological differences, such as differences in confidence and risk perception. These studies lend support to the arguments of Seifert et al [14] and Noakes [15] of athletes as complex adaptive systems whose behaviours are governed by the interplay between cognition and action, and who regulate their effort and energy expenditure according to the information available. As noted by Spindler and colleagues [32], there is a need for further research into the psychological factors governing success in elite cycling to improve understanding of the interplay between cognitive function, athlete behaviour, and competition performance.

#### Other Individual Features

The effects of nutrition and ergogenic aids on performance have received some attention in the literature [73]. No studies appear to have been performed to establish the efficacy of these strategies in actual competitions.

Performance improvements in solo time-trial events have been attributed to advances in bike technology and aerodynamic positioning [31], but in races where competitors are able to draft, the benefits of technology are much more difficult to quantify. Lippi and colleagues [54] analysed the average speed of Tour de France winners across the decades and attributed some of the improvement to major advances in bike technology from 1926 to 1970, as well as improvements in training, nutrition, and an increasing prevalence of doping across this period (see ‘Subversive Behaviours: the Prevalence of Doping’ section). The stabilisation of average speeds of Tour de France winners in recent decades is thought to be a result of reaching a ceiling in improvements in training and bike technology, as well as improvements in anti-doping measures. Notably, the improvements in cycling performance attributed (in part) to advances in bike technology by Haake [31] and Lippe et al. [54] occurred over several decades, whereas most athletes competing against each other in any particular race or competition are likely to have access to technological developments at roughly the same time. As such, differences in bike technology in races with multiple opponents are unlikely to be of a magnitude that differentiates between cyclists’ chances of success within a particular race.

Nationality or regional origin has been shown to influence performance and chances of success, with riders and teams seemingly more willing to take risks or expend effort when races hold some kind of national significance or are located closer to their country of origin [67] (see also ‘History and Prestige’ section). In downhill mountain biking, skill level had a positive effect on a rider’s self-confidence and their subsequent performance, but no significant relationship was found between performance and past experience, or performance and access to quality equipment [26]. Similarly, in track and road cycling, increased elite competitive cycling experience did not correlate with better performance [42, 67], but increased competition exposure in the *developmental years* was more predictive of future success than physiological measures for road cyclists at World Tour level [27]. There is also some evidence to suggest a male cyclist’s physical attractiveness, masculinity and likeability is linked to performance and chances of success in professional road cycling [80, 88]. A cyclist’s willingness to abide by the social norms that govern interactions between riders in the peloton can also influence their likelihood of success [36] (see ‘Social Mechanisms: Unofficial Norms and Peloton Sub-Culture’ section). Finally, there is some evidence that an individual must have the right genetic make-up to succeed in elite cycling by responding appropriately to training stimuli [28]. Lack of consistent findings across these features means these results are suggestive of an effect rather than conclusive, and more research is required to establish the robustness of these findings and the magnitudes of their effects on the performance of elite cyclists in race events.

### The Tactical Dimension

In competitions where opponents are able to interact, riders continually adapt to the actions of opponents and the changing race environment [81, 89–91]. The tactical dimension refers to features arising from this interaction between competitors and the actions they take in response to what is occurring. Understanding the effects of inter-personal dynamics on cyclists’ behavioural and tactical decisions is important if we are to make sense of their performances in events other than solo time trials.

#### Drafting Features: Pacing, Positioning and the Emergence of Pelotons

In race events, the presence of other cyclists enables drafting to occur, whereby a cyclist can reduce the energy cost of maintaining a particular speed by riding in the slipstream of other cyclists [37, 71, 92, 93]. By working together and taking turns to ride in the lead position, the energetic benefits of drafting allow a group of cyclists to sustain a speed greater than a cyclist could sustain riding alone [93]. The term *peloton* describes a group of cyclists who ride as a pack or bunch, a form of cooperative behaviour that emerges due to the energetic benefits provided by drafting [85]. The drag experienced by cyclists in the mid to rear positions of large pelotons (~ 120 riders) is ~ 5 to 10% of that experienced by an isolated cyclist riding at the same speed, and ~ 16% of that experienced by the cyclist leading the peloton (who experiences a reduction in drag due to the ‘upstream’ flow disturbance caused by the mass of riders following behind) [94]. Positions towards the rear of the peloton provide greater energetic savings, but are tactically disadvantageous, as the risk of collisions increases and it is harder to manoeuvre past opponents when nearing the finish line, or to respond to or launch attacks (whereby riders attempt to break away from the front of the main peloton and ride in advance of their opponents despite the higher energetic cost) [61, 94]. Consequently, cyclists tend to compete for the drafting and positional resources available, and effective use of these two resources has been shown to affect a cyclist’s chances of success in racing [37]. In the elimination, a mass-start race in the track-cycling omnium, the most successful cyclists were shown to be those who rode towards the front of the peloton (tactically advantageous) and in positions lower on the track (energetically advantageous) throughout the race [41]. Menaspà and colleagues [34] presented a case study of a Tour de France rider who was able to improve his chances of success in the final sprint by spending less energy through effective positioning and drafting during the earlier stages of the race. In cyclo-cross, top-ranked cyclists expended more energy in the initial laps of a race in order to gain a positional advantage over their opponents, after which they settled into a more sustainable pace for the remaining laps [35].

#### Interpersonal Features: ‘Coopetition’, Cooperation and Defection

While riding in a peloton reduces the overall energy cost of maintaining a certain speed, it can also narrow the gap between cyclists’ physiological capacities [37]. A weaker cyclist who optimises use of the drafting resource through effective positioning could theoretically beat a stronger opponent who did not position well or regulate their energy expenditure as effectively [85]. As a result, a cyclist’s best course of action during a race is dependent on their individual characteristics (see ‘The Individual Dimension’ section), opponent characteristics and actions, contextual features of the race and the options available to them [39, 61]. To gain a positional advantage over their opponent(s), some cyclists will launch an attack and attempt to ride in advance of their opponent(s) [33, 36, 39]. Attacking cyclists often form into smaller groups termed ‘breakaways’, electing to share positional and drafting resources in order to reduce the energetic cost of staying ahead of the main peloton. The degree of cooperation displayed in a breakaway group appears to be influenced by the size and physiological heterogeneity of the riders in the group, with cooperation increasing as the breakaway group size decreases and the physiological disparity narrows amongst the riders [37]. If a cyclist in the breakaway refuses to cooperate in sharing the drafting resource with their opponent(s), the likelihood of the other riders defecting (not sharing in the workload) also increases, and the breakaway is likely to fail [36]. A number of researchers have used game theory to characterise the dilemma cyclists face between cooperating, attacking or defecting [39, 66, 85]. Mignot [85] characterised the dilemma as a zero-sum sequential-move game, where the optimal timing of an attack or breakaway attempt is the point at which delaying for any longer reduces the cyclist’s chances of winning the race. The option to cooperate, attack, or defect has also been characterised as a prisoner’s dilemma, where each cyclist has an incentive to be the first to defect [39, 66].

#### The Influence of Contextual Factors on Race Dynamics

Modelling work from a number of authors has highlighted how contextual features alter the best choice of action for a cyclist, breakaway group or peloton during a race and the influence of these features on the chances of a cyclist’s or breakaway’s success [13, 36]. For example, as the speed of the peloton increases and riders approach their maximal sustainable power, drafting becomes a more valuable resource. In contrast, when the finish line is approaching or the peloton encounters crosswinds (where the formation of echelons can cause splits in the field), increased value is placed on the more forward positions [38]. Olds [13] found that breakaway-group size, chase-group size, gradient, and remaining race distance all affected the velocity, time to exhaustion, and critical gap size a breakaway group needed to succeed. Agent-based modelling by Hoenigman and colleagues [36] indicated that stronger cyclists seem to benefit from adopting a strategy of cooperation, while weaker cyclists appear to be better off defecting. On a practical level, the influence of contextual factors on a rider’s best choice of action was demonstrated by the analyses of Moffatt and colleagues [40], who showed that distance to finish, physiological differences, and relative rider positioning during a race influenced the likely outcome of head-to-head match sprints in track cycling. In a similar analysis of sprint finishes of Tour de France stages, cyclists’ chances of winning were linked to the distance remaining, the number of teammates still riding in support, and the positioning of the cyclist in the final meters of the race [11]. In stage races, the chances of a breakaway succeeding were also dependent on the threat it posed to the leader in the general classification (overall cumulative position) and whether the energy expenditure required to chase down the breakaway was deemed supportable by the cyclists in the main peloton [39]. For example, if the next stage of the race was perceived to be tough, the riders often considered it more prudent to save their energy than catch the breakaway.

#### Team Dynamics

In professional road cycling, a cyclist’s overall performance is influenced by the skills and attributes of their teammates, as most riders work to improve their team leader’s chances of success rather than their own [56, 61, 67]. While an individual is awarded the win, their efforts are often aided by the work of teammates, who provide drafting and positional assistance to allow the team leader to conserve energy for the most crucial moments of the race [56, 61, 67]. Torgler [67] applied theories from labour economics to examine productivity in professional road cycling teams, finding that an individual cyclist’s performance suffered if they were in a team of high performers, as that rider was expected to sacrifice their own chances of success in order to improve the chances of their teammate(s). For the team leader, the benefit of having teammates in support was also demonstrated by Menaspa and colleagues [34], who found the chances of a cyclist winning a sprint stage of a Grand Tour increased when they had teammates providing a non-competitive drafting benefit during the last 60 s of the race. Features of team dynamics at the strategic level are explored in ‘Team Hierarchy: Opportunity’ section.

### The Strategic Dimension

Strategic features refer to elements of the competition or race environment that shape the decisions of a cyclist, team, or organisation before the competition begins and set their actions within a wider context [95]. Cycling differs from most sporting competition in that the features and format of cycling races are heterogeneous, with competition structures, course distances, routes and terrain differing from race-to-race. The multiple-prize reward structure in professional road cycling adds further complexity to the race environment, leading to the emergence of efficiency, non-competitive behaviour, and occasionally, the formation of tacit alliances and collusion between riders.

#### Competition Features: Terrain, Environment and Competition Structure

The format and structure of a cycling competition alters the technical-skill requirements and physiological characteristics associated with optimal performance, predisposing riders with particular characteristics to be better suited for certain competition formats or races [22, 41, 43, 47, 78, 82, 83]. Cycling competitions may consist of a single race or be multi-race, single day or multi-day competitions; take a mass-start, one-versus-one, individual or team format; take place indoors, outdoors, in purpose built facilities, off-road, on road or on purpose built tracks; and run across a range of surfaces and terrain [56]. Numerous studies have explored the attributes required for success across the various formats of cycling competition, demonstrating that each presents different metabolic, physical, physiological, technical and cognitive stressors on an individual [5, 21, 24–26, 29, 41, 43, 68, 72]. In this sense, the attributes required for optimal performance in any particular cycling discipline, event, or race are highly dependent on the competition features. Cyclists therefore tend to specialise into particular disciplines that suit their characteristics and improve their likelihood of success, as detailed in ‘Morphological Features’ section.

The structure of a competition has also been shown to alter the physiological characteristics associated with success [46, 74]. Rodriquez-Marroyo and colleagues [23] demonstrated the impact of competition structure on performance, detailing the changes in the workload demands on professional cyclists across 5-, 8-, and 21-day stage races. Adjustments to the format of the omnium, a 2-day competition in track cycling, have also shown the effect of competition structure on performance, with the addition of the elimination event altering the type of rider likely to do well in the overall competition [44]. Course route, commonly referred to as the *parcours* in road cycling*,* also influences cyclist performance. For example, race distance and difficulty and location of technical features (such as the inclusion of cobblestone sections, or position, gradient and number of hill climbs) all alter the physiological and technical demands on a rider [22]. While the competition format and route are fixed in advance, environmental (e.g., weather) conditions can also alter performance and likelihood of success. Wind and rain in particular can alter the value of the drafting and positional resources, altering the perceived risk associated with particular racing strategies and the way cyclists elect to expend their energy [K.E. Phillips, unpublished observations].

#### Competition Calendar Effects: the Emergence of Efficiency and Non-Competitive Behaviour

Competition features have been shown not only to influence the *type* of cyclists likely to succeed, but the *way* cyclists compete, particularly in competitions consisting of a series of races, such as the Match Sprint, Keirin and Omnium tournaments in track cycling, and multi-day stage racing in road cycling. In multi-race or multi-stage contests, cyclists must balance their desired goals against the efforts required to achieve them, resulting in the emergence of strategies related to efficiency, whereby riders seek to optimise performance across a series of races to secure a larger objective [52]. For example, road cyclists aiming to win the general classification in a multi-day stage race often adopt conservative racing strategies in the early stages, saving energy for the critical moments of the competition [85]. The potential payoffs of using a strategy of efficiency have been shown in the track cycling Omnium, where a cyclist aiming for the overall title is able to finish as low as 6th place in one of the events and still be in contention for the overall title [44]. The importance of efficiency in multi-stage cycling races can also lead to the emergence of non-competitive behaviour, where cyclists who are unlikely to win a race or secure a high ranking in the various classifications have a higher incentive to abandon the race, should it not go as they planned [67]. Abandoning a race is a rational way for a cyclist to conserve energy, reducing the cost of the current effort to protect performance in future events [67].

#### The Influence of Objectives: Incentives and Rewards

The diversity of racing objectives amongst cyclists presents a unique challenge for researchers seeking to understand the performance of individual cyclists in real-world competition, namely that numerous competitors may deliberately withhold their best efforts [52]. Instead, riders may be motivated by objectives that have nothing to do with winning a particular race or stage [52]. For example, elite track cyclists have discussed employing conservative racing strategies, taking less risks and seeking only to finish above a certain rank in order to secure a qualification spot for a more prestigious race, such as the Olympic Games (K.E. Phillips, unpublished observations). In professional road cycling, the multiple-prize structure of the competition shapes cyclists’ goals and thereby influences their choice of action in any given race or stage [85]. Cyclists may be focused on securing a classification title (e.g., general, mountain, points, young rider) or focused on gaining TV exposure for sponsors or their personal brand by being in the breakaway group at the front of the race [52].

An individual’s or team’s *perception* of their chances of success in a competition is known to influence their selection of race objective, affecting their selection of racing strategy and willingness to expend effort. For example, professional road cycling teams have been shown to adjust their objectives depending on the characteristics of the riders in a given event, stage, or competition [55]. A team may also elect to adjust their objective mid-competition, seeking a secondary prize (such as a stage win or other classification) owing to an early injury, change in leadership, crashes, or a change in their perceived chances of success [52].

#### Collusion and Association

Race outcomes in cycling are sometimes influenced by collusion, when opponents deliberately cooperate, form tacit alliances, or swap monetary compensation in return for assistance in achieving a goal [56, 64]. The cooperation seen between opponents in cycling (as referred to in ‘Competition Calendar Effects: the Emergence of Efficiency and Non-Competitive Behaviour’ section) is an interesting case study for game theorists, as cooperation violates the implicit assumptions of the zero-sum game (one side wins, one side loses) that characterises most sporting competitions [85]. In cycling, one competitor may ‘carry’ another competitor, even to the point of helping the other to win, with the goal of securing a larger objective [65]. Unlike in many other sports, conflict and association exist together in cycling.

On one hand, the theme of individualism reflects the unambiguous aspects of conflict, emphasizing individual or team effort that occurs within the framework of the basic rules and results in a win or loss. On the other hand, a theme of collectivism has emerged, reflecting some of the situational particularities of the sport that require an association between opponents, called ‘drafting’. Albert [65] p. 344

Riders have been known to collude or ‘fix’ the outcome of races, offering some compensation in return for the assistance of other riders [61, 64]. Mignot [61] uses the example of Miguel Indurain and Claudio Chiappucci in the 13th stage of the 1991 Tour de France to illustrate fixing, where Indurain allowed Chiappucci to win the stage on the premise that he contributed to the workload of the breakaway group, helping ensure the group stayed clear of the main peloton and enabling Indurain to achieve his aim of securing a classification jersey.

#### Team Hierarchy: Opportunity

Researchers seeking to examine the performance of cyclists in real-world competition must also understand that a rider’s performance may depend on whether they are given the opportunity to race for the win. Professional road cyclists who are not team leaders often have two conflicting goals: seeking to contribute to their team success while on a secondary level aiming to contribute to their own personal success and career longevity [49]. Professional road cycling teams may consist of up to 30 riders, with only eight or nine of these riders permitted to start in Grand Tours, and entries are also limited in other ProTour events. Team management will normally select a team and a ‘lead’ rider based on the characteristics of their riders relevant for the race features and importance [47]. Team leaders are generally more successful than other cyclists across a racing season, but whether this is due to the team leader’s superior individual characteristics or because they benefit from the work of their teammates is unclear [49]. Cyclists riding in support of their team leader (termed ‘domestiques’) have little incentive to continue racing once they have fulfilled their expected duties. Domestiques are known to ‘sit up’ once they have executed their support task(s), no longer seeking to remain competitive and only seeking to finish the race within the time cut-off to ensure they are permitted to start the following stage [K.E. Phillips, unpublished observations].

### The Global Dimension

Elite cycling competitions take place within a complex social and organisational setting, where economic, socio-cultural and historical forces shape behaviour and decisions made by governing bodies, race organisers, cycling teams, and the cyclists themselves [96]. Researchers in the fields of economics, management and sociology have provided some insight into how organisational changes and reforms, largely driven by attempts to improve the globalisation and professionalism of the sport, have impacted the performance of cyclists in recent decades [49, 51, 64, 66, 69, 96]. To date, authors have focused almost exclusively on male professional road cycling.

#### Economic Features: Revenue Generation, TV Rights, Sponsorship

Various authors have explored the economic drivers of male professional road racing, detailing how the competing interests of multiple stakeholders shape the structure of professional racing and influence the competitive pressures on teams and riders [64, 66, 96]. As the primary governing body, the UCI formulates (and adapts) the rules of racing, regulates the classification of races and points ranking systems, and issues racing licenses to teams and riders. Organisational changes and reforms over the decades, designed by the UCI to improve the globalisation and professionalism of the sport, have led to major changes in racing behaviour by cyclists and their teams (see ‘Authoritarian Features: Governing Bodies and Race Organizers’ section) [96]. Race organisers also play a key role in shaping the structure of professional road racing, with many of the most prestigious events controlled by private corporations such as the Amaury Sports Organisation (ASO) [96]. Race organisers are known to alter the design of a race route to ensure high competitive intensity between riders, with the aim of increasing spectator interest to generate revenue, attract sponsors, and secure interest in the purchasing of TV rights [52, 97, 98]. External stakeholders, such as team sponsors and the media, also have a strong interest in ensuring the attractiveness of the contest, driven by a desire to increase TV viewership and ensure brand visibility [52]. As professional race teams are funded almost entirely from sponsorship, team managers have a strong incentive to promote sponsors’ visibility during a race and deliver race results, in order to ensure the ongoing financial viability of a team [96]. For riders, professional race contracts are often short, and while riders can participate in races only as part of a team, there is also a strong incentive for individual cyclists to perform well in order to secure a contract [50]. Aubel [57] argues that there is a potential link between the economic position of a team or rider and doping, reporting sanctioned riders were 5.8 times more likely to have experienced career interruptions or 61% more likely to have had multiple team changes (see ‘Subversive Behaviours: the Prevalence of Doping’ section).

Similar economic drivers of revenue generation via spectator engagement appear to be responsible for reforms made to other disciplines of cycling, namely BMX and track cycling (see ‘Authoritarian Features: Governing Bodies and Race Organizers’ section). For example, the removal of the solo time-trial events from the Olympic track-cycling program and introduction of the 2-day multi-race Omnium competition were seen as a reflection of the UCI and IOC’s desire to deliver more engaging and tightly fought contests for spectators [45].

#### Authoritarian Features: Governing Bodies and Race Organisers

Governing bodies and race organisers are known to implement changes to the regulations of cycling competitions in attempts to improve the competitive intensity of racing and thereby increase public interest in cycling [52, 61]. Reforms made to rules and racing regulations over the decades have allowed researchers to observe and analyse the subsequent changes in racing behaviour and performance [48, 58, 96].

A particularly influential reform was the introduction of a ranking system by the UCI in 1984, which was designed in part to increase competition and reduce collusion amongst professional road racing riders and teams [56]. UCI ranking points had a major influence on the way teams and riders prepared and raced [52, 56, 64, 96]. In races that had traditionally been fixed or used for training, teams became competitive, needing to accumulate ranking points across a wider number of races throughout the competitive season to gain entry to the more prestigious events [64, 96]. A secondary aim of the UCI was to globalise cycling, and the ranking system ensured teams competed in races outside of Europe, the traditional stronghold of the sport [52]. Brewer [64] described how the increase in competitiveness resulted in an effective lengthening of the competitive racing season and modified the pressures on teams and riders. For example, team managers were compelled to build a squad of cyclists capable of success across a wider range of races, rather than the more traditional method of structuring a team around the support of one key rider for the more prestigious event(s) [64]. For the cyclists, a higher expectation to perform well in races across the full competitive season increased the pressure to remain in top physical condition, driving advances in training techniques and technology, as well as inadvertently encouraging subversive behaviours such as doping [56, 64] (see ‘Subversive Behaviours: the Prevalence of Doping’ section).

Another influential reform was the introduction of a ban by the UCI on the use of radio technology in some professional road races from 2011 to 2015, which was also seen as an attempt to improve the competitive intensity of the sport and prevent racing from becoming too predictable [48]. Larson and Maxcy [48] found the change in information flow caused by the radio ban affected race structure and outcome, although they could not determine conclusively whether changes in the likelihood of breakaway success were due to absence of radio communications or changes in the cycling sub-culture across the period examined (see ‘Social Mechanisms: Unofficial Norms and Peloton Sub-Culture’ section). As a side note, the authors found the use of radio technology and likelihood of breakaway success were strongly modified by terrain variables (see ‘Competition Features: Terrain, Environment, and Competition Structure’ section).

Within a particular country, the regulations of national governing bodies impact the development, recruitment, and performances of elite cyclists. For example, Zheng [58] provided an interesting commentary on the relative underperformance of Chinese cyclists in Olympic and UCI ProTour cycling events, suggesting the under-representation of Chinese cyclists was due to organisational elements of the Chinese sporting system. In comparison, a strong talent development program within the Australian national system was apparently responsible for a 50% increase in the conversion rate of junior world championship athletes to success in the senior divisions [63].

#### Reward Mechanisms: Multiple Prize Incentives

The introduction of secondary prizes in the Tour de France by the ASO is an example of reward mechanisms used to encourage cyclists to compete and create a dynamic spectacle that will draw public interest [61, 69]. Secondary prizes, such as time bonuses, intermediate sprints, and combative rider prizes, provide an incentive to cyclists who are not in contention for the overall win, by presenting them with opportunities to gain ranking points or media exposure while adding to spectator interest [98]. As riders vying for the overall win in multi-day stage races are likely to adopt strategies of energy conservation and non-competitiveness in the early or flat stages, secondary prizes help maintain a level of competitive intensity by incentivising the other riders.

Formal rules and regulations can also inadvertently provide a disincentive for teams and riders to compete. Rebeggiani and Tondani [56] outlined how the UCI inadvertently encouraged non-competitive behaviour from professional teams across the racing season by restricting the total number of professional road teams in the UCI ProTour, thereby making it a closed league. Without the risk of relegation to a lower league, teams were concentrating their best efforts on races organised in their sponsors’ home countries or on only a few other races each season. The authors argued the UCI would better achieve their aim of improving the competitive intensity of racing by opening the league and increasing the number of ProTour teams, thereby providing an incentive to compete in races teams may otherwise not target. The UCI has adopted a version of this recommendation in recent years [99].

#### Social Mechanisms: Unofficial Norms and Peloton Sub-Culture

In addition to the formal rules governing the competition, a cyclist’s behaviour and decisions are constrained by unofficial norms or social mechanisms that exist within the peloton [65, 69]. Unofficial norms emerge when it is beneficial for race participants to enforce a subset of social rules, driven by a collective desire to ensure the profitability of the sport [62]. Unofficial norms dictate the shared expectations of how cyclists should behave within the peloton and individuals who do not abide by these social expectations are subjected to sanctions by other members of the peloton [62, 69]. For example, if a race favourite or race leader should suffer a mechanical issue, the unofficial norm dictates that the other cyclists should not make use of the opportunity to benefit themselves by making an attacking move [62, 85]. Cyclists who disobey this norm are often punished by the peloton, who collectively refuse to cooperate with the ‘defector’, denying them TV exposure (e.g., by chasing down any breakaway attempt), and in more extreme cases, by physically interfering with the defectors’ ability to ride [62]. Albert [65] conducted a sociological examination of the peloton to explore the influence of social dynamics on the performance of cyclists in road races, seeking to understand why riders share the lead, why a peloton may allow a breakaway to form, and why some breakaways succeed when others fail. The author concluded that using formal rules to explain the constraints on cyclist behaviour is insufficient to capture the experienced reality of the sport, arguing that the informal social norms must be considered if we are to understand racing behaviour. Fink and Smith [62] built on this work by examining the unofficial norms that govern behaviour in elite road cycling and by explaining how these norms affect the profitability of the Tour de France for race organisers and maintain the attractiveness of the event for spectators.

#### Subversive Behaviours: the Prevalence of Doping

Cycling has been characterised by numerous doping scandals and the phenomenon of doping, and its effects on cycling have been examined from a number of angles [53, 54, 57, 59, 64]. The influence of doping on the performance of elite cyclists is difficult to quantify because there is no accurate way of knowing who doped, to what extent, and when [98]. To examine the potential influence of doping on race performance and assess the effectiveness of anti-doping measures, some researchers have used secondary measures of performance, such as changes in the average speed of Grand Tour winners across the decades [53, 54]. The decrease (or plateau) in average speed of Grand Tour winners since the early 2000’s has been attributed to strengthened anti-doping measures [54, 100], but changes in the socio-cultural environment in which riders compete provide equally plausible explanations [59, 64]. Such changes include modification of team structure, sponsorship, inter-team dynamics and rider preparation [59, 64, 96].

Fink and Smith [62] outlined how the challenges associated with monitoring and preventing doping amongst cyclists at an organisational level resulted in specific social norms developing amongst teams and riders. As doping behaviours were not able to be observed directly, cyclists were uncertain who was doping, so the ‘clean’ cyclists could not collectively monitor and punish those using illicit substances [64]. Instead, a social norm known within the sport as the *omerta* developed, where organisers and riders both accepted doping was prevalent, but the established social norm was not to discuss it publicly, in order to protect the sport and the revenue it generated [62, 64]. Economists have used game theory to explain how teams and riders rationalised doping, where the economic and financial benefits of improved performance, along with prohibitively high sanctioning costs, resulted in a Nash equilibrium in which it was most profitable for all cyclists to dope [62, 98]. Brewer [64] drew links between authoritarian, reward, and social mechanisms and the increasing incentive to dope, particularly for teams at risk of losing sponsorship or for riders at risk of not securing an ongoing professional contract. Aubel et al. [57] found a higher risk of doping amongst cyclists who began their professional careers before 2005, but noted the reasons for the declining number of sanctioned riders since 2005 were ambiguous. The reduction could be due to improved anti-doping measures, including the introduction of the biological passport in 2008, changes to the structure of the World Tour, changes to the socio-cultural norms of the peloton, or ‘improvements’ in the doping practices of teams and athletes making the use of banned performance enhancing substances harder to detect.

#### History and Prestige

Brewer [64] and Mignot [61] provided thorough overviews of the history of professional road cycling and explored how changes in global mechanisms across the decades have led to changes in rider performance. As outlined in ‘Economic Features: Revenue Generation, TV Rights, Sponsorship’ and ‘Subversive Behaviours: the Prevalence of Doping’ sections, changes made by governing bodies aimed at increasing the commercialisation and globalisation of the sport altered the pressures on team managers and riders, leading to changes in team organisation and rider preparation, fostering changes in the social dynamics of the sport and inadvertently resulting in the rationalisation of doping practices. The growth of commercial sponsors led to increased professionalism, enabling riders to increasingly specialise as the racing calendar gradually increased in length and intensity [64]. Furthermore, the roster of team riders shifted from being organised around the support of a single team leader to an organisation of sub-teams that trained for peak performance in specific races or Grand Tours [61]. Team managers started selecting and organising sub-groups of riders from their team roster for success in particular races, targeting either a Grand Tour*,* the Classics (Milan-San Remo, Tour of Flanders, Paris-Roubaix, Liège-Bastogne-Liège and Tour of Lombardy) or races with regional or national significance [61]. Top cyclists and their support riders then adjust their training and periodisation to peak for these particular events [56, 97]. While evidence of improvements in cyclist performance due to race prestige remains predominantly anecdotal, teams certainly appear to place increased importance on performance in particular races and tours [61, 64]. In professional road cycling, the Tour de France remains the most prestigious competition, while in other cycling disciplines the World Championships are considered the most prestigious event in non-Olympic years [61].

#### Other Societal and Organisational Features

The preceding sections of this review have dealt with performance of individual cyclists. There are only a few cycling disciplines where team performance is of interest, and in professional road cycling there has been only one publication addressing the determinants of team performance. Prinz and Wicker [60] applied concepts from management research and labour markets to assess the effect of team composition on the performance of professional road cycling teams in the Tour de France. Having a diverse range of tenure (length of time in team) was positively associated with team performance, which the authors suggested was due to internal competition for selection between team riders raising performance standards or preventing stagnation in those with longer careers. Age diversity of team riders had a positive (albeit non-significant) effect on performance, while diversity in nationality and language had little effect. Another explanation for these relationships is that early-career riders will join any team, but as their performance improves, they are likely to move to teams where they have more opportunities. Prinz and Wicker also found that a wide range of body mass index (BMI) scores was linked to poorer team performance, likely due to overall performance indices favouring hill climbers, who generally have lower BMI. Previous Tour de France participation and previous stage wins were not significantly related to team performance, but the number of riders finishing the Tour de France did matter, which likely reflects the sharing of workload between team members. The authors concluded that team managers seeking team success needed to consider the composition of their teams, selecting riders capable of finishing the race and with a diverse range of tenure [60].

### Limitations

As outlined in ‘Inclusion Criteria’ section, a narrative-synthesis approach was used to systematically review the selected articles and formulate dimensions and features. Of the articles that met the inclusion criteria, a majority analysed the competition performances of road cyclists, and in particular, male professional road cyclists. Consequently, some of the dimensions and features identified in this article are based on research in a single cycling discipline (see Table 1 for detail). There may also be factors affecting the performance of cyclists that are being used in competition but that have not been the subject of published research; for example, teams who have developed new ergogenic aids or improvements in technology are unlikely to publish their findings. Finally, several features known to enhance cyclists’ performance in non-competition settings have received limited attention in this review because the extent of their implementation and effects in actual competitions are unknown.

## Conclusions

The aim of the current study was to improve our understanding of how performance emerges in elite cycling competitions, with a particular focus on the effects of contextual factors, presence of and interaction with opponents, environmental conditions, competition structure, and socio-cultural, economic and authoritarian mechanisms. The challenges associated with modelling the performance of cyclists in the complex environments that define cycling racing become evident when the features and dimensions influencing race performance are collated. In particular, there are limitations in using a traditional reductionist approach to understanding the performance of cyclists in elite racing. Sports performance research needs to be 'holistic, idiographic and take a process-oriented approach' that emphasises 'the analysis of emergent patterns of coordination and control underpinning performance' [9, 16, 101–103]. Moving forward, the challenge is for sports performance researchers to find methodologies and techniques that enable elements of performance to be considered in concert rather than in isolation, and for the complex interplay and interactions between dimensions and features of racing to be better understood. We must search for data collection and analysis techniques that allow us to adequately account for and explain the interactions and mechanisms underpinning successful cycling performance in racing contests [7–10, 100, 104].

The breadth of scientific disciplines encompassed in the present review provides some insight into how combining approaches, by using mixed methods and/or interdisciplinary approaches, would enable ‘multiple valid accounts of a phenomenon’ by improving trustworthiness, dependability, confirmability, transferability and authenticity in research [105]. There is a place for quantitative laboratory-based research that examines specific elements of performance, just as there is equally a place for qualitative investigations exploring the nuances and meanings that shape the decisions and actions of athletes in competition [20].

To be competitive at the elite level, a cyclist needs a high level of physiological fitness, but team managers, coaches and athletes seeking to improve performance should give attention to features beyond those of the individual. Competitive performance is also constrained by tactical features emerging from the inter-personal dynamics between cyclists, strategic features related to the competition and global features related to the organisation of the sport.

## Data Availability

Not applicable

## Abbreviations

ASO: Amaury Sports Organisation
BMI: Body mass index
UCI: Union Cycliste Internationale
